# Transgenic *C. elegans* Dauer Larvae Expressing Hookworm Phospho Null DAF-16/FoxO Exit Dauer

**DOI:** 10.1371/journal.pone.0025996

**Published:** 2011-10-07

**Authors:** Verena Gelmedin, Thomas Brodigan, Xin Gao, Michael Krause, Zhu Wang, John M. Hawdon

**Affiliations:** 1 Department of Microbiology, Immunology and Tropical Medicine, George Washington University Medical Center, Washington, D. C., United States of America; 2 Laboratory of Molecular Biology, National Institute of Diabetes and Digestive and Kidney Diseases, National Institutes of Health, Bethesda, Maryland, United States of America; 3 Department of Pharmacology, UT Southwestern Medical School, Dallas, Texas, United States of America; Ecole Normale Supérieure de Lyon, France

## Abstract

Parasitic hookworms and the free-living model nematode *Caenorhabtidis elegans* share a developmental arrested stage, called the dauer stage in *C. elegans* and the infective third-stage larva (L3) in hookworms. One of the key transcription factors that regulate entrance to and exit from developmental arrest is the forkhead transcription factor DAF-16/FoxO. During the dauer stage, DAF-16 is activated and localized in the nucleus. DAF-16 is negatively regulated by phosphorylation by the upstream kinase AKT, which causes DAF-16 to localize out of the nucleus and the worm to exit from dauer. DAF-16 is conserved in hookworms, and hypothesized to control recovery from L3 arrest during infection. Lacking reverse genetic techniques for use in hookworms, we used *C. elegans* complementation assays to investigate the function of *Ancylostoma caninum* DAF-16 during entrance and exit from L3 developmental arrest. We performed dauer switching assays and observed the restoration of the dauer phenotype when *Ac*-DAF-16 was expressed in temperature-sensitive dauer defective *C. elegans daf-2(e1370);daf-16(mu86)* mutants. AKT phosphorylation site mutants of *Ac*-DAF-16 were also able to restore the dauer phenotype, but surprisingly allowed dauer exit when temperatures were lowered. We used fluorescence microscopy to localize DAF-16 during dauer and exit from dauer in *C. elegans* DAF-16 mutant worms expressing *Ac*-DAF-16, and found that *Ac*-DAF-16 exited the nucleus during dauer exit. Surprisingly, *Ac*-DAF-16 with mutated AKT phosphorylation sites also exited the nucleus during dauer exit. Our results suggest that another mechanism may be involved in the regulation DAF-16 nuclear localization during recovery from developmental arrest.

## Introduction

The insulin/insulin growth factor (IIS) pathway is involved in embryogenesis [1], cell differentiation [2], development, and aging [3], [4] in diverse species. In the free-living nematode *Caenorhabditis elegans*, this pathway mediates entry into and exit from the developmentally arrested dauer stage by negatively regulating the activity of a FoxO-family forkhead transcription factor DAF-16 [5]–[10]. The developmentally arrested, resistant dauer stage allows *C. elegans* to survive in unfavorable environments for several months after its second molt [11]. In *C. elegans*, DAF-16 localizes to the nucleus under dauer-inducing conditions, where it binds to promoter regions of target genes that induce and maintain dauer [8], [12]. In responses to IIS, DAF-16 is phosphorylated by the activated serine-threonine protein kinase AKT/protein kinase B (AKT/PKB), therefore creating binding sites for 14-3-3 proteins (FTT). Interaction between FTT and phosphorylated DAF-16 results in its nuclear exclusion and cytoplasmic retention leading to reproductive growth [13], [14].

The free-living infective third stage larvae (L3) of parasitic nematodes, such as hookworm, are biologically and functionally analogous to the *C. elegans* dauer stage [15]–[17]. During infection, hookworm L3 encounter a signal in the host that activates suspended developmental pathways that lead to resumption of development and progression to the L4 and adult stages. This is exactly analogous to recovery from dauer in response to environmental cues in *C. elegans*. Therefore, the “dauer parasitism hypothesis” proposes that common molecular mechanisms mediate both the resumption of development by hookworms during infection of the host, and recovery from the dauer stage in response to improved environmental conditions in *C. elegans* [17]–[19].

The inability to resume development *in vitro* necessitates maintaining hookworms in an animal host, which precludes the development of genetic systems. Consequently, little is known about the molecular events of early infection. What is known is centered on the larval activation process, in which L3 can be induced to resume feeding and secrete infection-associated molecules *in vitro* [20]–[25]. Evidence indicates that activation is regulated by IIS and DAF-16 in hookworms. Orthologs of *C. elegans* DAF-16 and 14-3-3, *Ac*-DAF-16 and *Ac*-FTT-2, have been identified recently from the hookworm *Ancylostoma caninum* [26], [27]. *Ac*-DAF-16 contains a highly conserved forkhead DNA-binding domain and three potential AKT phosphorylation sites (S107, T312, and S381). Recombinant *Ac*-DAF-16 bound to and drove transcription from a consensus binding element found in the promoters of FoxO/DAF-16 target genes from *C. elegans* and mammals [26]. *Ac*-DAF-16 also bound recombinant *Ac*-FTT-2, an interaction that required intact AKT phosphorylation sites [27]. These experiments indicated that *Ac*-DAF-16 is a functioning transcription factor and further support that IIS pathway plays a critical role for hookworm L3 re-activation during infection.

In the present study, we used *in vitro* and heterologous systems to investigate the mechanism of DAF-16 action. We demonstrate that the predicted sites on *Ac*-DAF-16 are phosphorylated by AKT *in vitro*. Using cell culture, we show that *Ac*-DAF-16 is negatively regulated by IIS, and that *Ac*-DAF-16 is exported from the nucleus in response to IIS. Finally, using transgenic *C. elegans*, we demonstrate that *Ac*-DAF-16 partially complements *daf-16* loss of function mutations to restore dauer formation. Our studies lend support for the dauer parasitism hypothesis, and indicate that the IIS pathway is important, but not exclusively responsible for the regulation of recovery from the arrested L3 via *Ac*-DAF-16.

## Materials and Methods

### Ethics statement

This study was carried out in strict accordance with the recommendations in the Guide for the Care and Use of Laboratory Animals of the National Institutes of Health. The protocol was approved by the George Washington University Medical Center Institutional Animal Care and Use Committee (protocol number: A147).

### *In vitro* phosphorylation assay

*Ac*-DAF-16 has three potential AKT phosphorylation sites base on primary amino acid sequence analysis. To determine if AKT phosphorylated *Ac*-DAF-16 on the predicted sites, 2 µg of recombinant human AKT (Upstate) were incubated with 1.25 mM rATP (Sigma-Aldrich), 2 µg *Ac*-DAF-16-peptide and 30 µl kinase buffer from ADP Quest System (Discoverx) for 1 h at 30°C. Three DAF-16 peptides of 15 amino acids length containing the putative AKT phosphorylation sites were used as substrates. Crosstide was used as substrate in the positive control, and reactions with phosphorylated peptides and without AKT and rATP served as negative controls. The kinase reactions were stopped by adding ADP detection reagents A and B, and the fluorescent light emission was determined after 30 minutes at RT in a 96-well plate reader (DTX Multimode detector Biomek FX/NX, Beckman Coulter, CA, USA) according the manufacturer's instructions. ADP concentrations proportional to the light emission were calculated from an ADP standard curve. The kinase reactions were also analysed by Western blotting using phospho-specific rabbit *Ac*-DAF-16-peptide antibodies following SDS-PAGE as described below.

### Luciferase assay

In response to IIS, *C. elegans* DAF-16/FoxO is phosphorylated by AKT. To determine if *Ac*-DAF-16 was phosphorylated in response to IIS, HepG2 cells were co-transfected with pCMV4-*Ac-*DAF-16 wildtype or phosphorylation site mutant constructs (single mutants, S107A, T312A, S381A; double mutants, S107A/T312A; S107A/S381A, T312A/S381A; and triple mutant, S107A/T312A/S381A) [27], the luciferase reporter vector p6xDBE-*luc* containing 6 copies of the canonical DBE upstream of firefly luciferase [26] and a *Renilla* luciferase reporter plasmid as an internal control. Cells co-transfected with pGL3 vector (Promega) with intrinsic promoter activity and *Renilla* reporter plasmid served as positive control. Transfections were performed in 24 well plates according to the Genporter3000 protocol (Genlantis). The cells were grown in Dulbecco's Modification of Eagle's medium (DMEM; Cellgro) supplemented with 10% FBS, 100 U/ml Penicillin and 0.1 mg/ml Streptomycin (Cellgro) for 24 h. After incubation, the cells were starved in DMEM without FBS for 16 h and treated with insulin at a final concentration of 10 ng/ml for 1 h. Finally, the cells were treated with 100 µM AKT inhibitor IV, AKT inhibitor IX and LY294002 (Calbiochem) for another hour. Control cells were treated with solvent alone (0.1% DMSO). The cells were washed and the luciferase activities measured with a Sirius luminometer using the Dual-Glo Luciferase Assay system (Promega). Treatments were performed in triplicates. The ratio of firefly to *Renilla* luciferase were calculated and the mean and SD determined. The experiment was repeated three times.

### Cell fractionation and Western blot

The mammalian cell line HEK293 was transfected with a construct encoding full-length wildtype *Ac-*DAF-16 (clone Daf16.4Ba.pCMV.2J, [26]) using the lipotransfection reagent Genporter3000 (Genlantis) in 6 well plates according to the manufacturer's instructions. After 48 h in DMEM with 10% FBS (Gibco), the cells were starved for 24 h in 2 ml medium without FBS. A mock transfection of HEK293 with the empty pCMV-Tag4 vector served as a control. After treatment, the cells were washed three times with 1x PBS-Tween 0.05% (PBS-T) and fractionated using the Qproteome cell compartment kit (Qiagen). Following acetone precipitation, the protein fractions were separated by SDS-PAGE and transferred to nitrocellulose membranes. The membranes were incubated in 5% skim milk in 1x PBS-T and subsequently probed with mouse anti-GAPDH (1∶2,000 Abcam), mouse anti-histone (1∶500) (Chemicon International) or the rabbit DAF-16 anti-serum (1∶20,000) for 16 h at 4°C with shaking. After three washes in 1x PBS-T, the membranes were incubated with the HRP-conjugated anti-mouse (1∶10,000) or anti-rabbit (1∶5,000) secondary antibody for 1 h at RT. The washing steps were repeated and the membranes exposed to X-ray film following incubation with ECL chemiluminescence reagent (Pierce) for visualization.

### Genetic stocks of *C. elegans* and transformation constructs

The *C. elegans* double mutant strain *daf-2(e1370);daf-16(mu86)* was used as the parent strain for microinjection and as control in dauer assays. The control strain *daf-2(e1370)* was obtained from the *Caenorhabditis* Genetics Center (University of Minnesota). The third control strain, CF1449, was a *daf-2(e1370);daf-16(mu86)* double mutant carrying the transgene construct encoding an N-terminal fusion of GFP and wildtype *C. elegans* DAF-16 downstream of the daf-16α promoter was kindly provided by Cynthia Kenyon [28]. All strains were maintained on *E. coli* OP50 growing on NGM plates at 16°C according to standard methods. For the complementation experiments, the coding region of wildtype *Ac-daf-16* (GenBank accession number ACD85816) [29] was initially cloned into pWZ128 with *pdpy-30* as the promoter. However, several of the transgenic lines were unstable. Therefore, the coding region of *Ac-daf-16* was switched into pPV207 to create pWZ401 containing the *C. elegans daf-16α* promoter upstream of *Ac-daf-16*. In a further step the coding region for GFP was added at the end of the *Ac-daf-16* ORF leading to C-terminal GFP fusion protein. Mutant plasmids of *Ac-daf-16* (single mutants, S107A, T312A, S381A; double mutants, S107A/T312A; S107A/S381A, T312A/S381A; and triple mutant, S107A/T312A/S381A) were described previously [27]. All constructs were confirmed by DNA sequencing.

### Establishment of *C. elegans* transgenic lines and complementation assays

Constructs of the transgenes to be tested for rescue were mixed with marker plasmid (encoding *rol-6*) at equal volume, both at 100 ng/µl, and injected into gonads of young hermaphrodites of double mutant strain *daf-2(e1370);daf-16(mu86)*. Microinjected animals were reared and screened for transformed F1 progeny based on the roller phenotype. Positive F1 progeny were re-plated and roller F2 progeny selected again. Positive F2 generation and beyond were propagated as transgenic lines. Two to six lines per transgene were obtained.

For dauer switching assays in *C. elegans,* egg-laying hermaphrodites from each transgenic line and control strains were placed on NGM plates seeded with *E. coli* OP50 lawns for 3-4 h at RT and subsequently removed. The plates were incubated at 25°C and after 96 h animals were scored. For the dauer rescue assay, the hermaphrodites were sustained on plates. After 96 h, dauer larvae were isolated by incubation in 1% SDS [11] and transferred to NGM OP50 plates. The plates were incubated at 16°C and observed daily for 7 days. The number of worms that developed were enumerated daily, and subsequently removed from the plate before they reproduced. Worms that failed to develop or died were also noted. Dauers isolated from the *daf-2(e1370*) strain and from *daf-2;daf-16* expressing *Ce*-DAF-16 were used as controls. All assays were conducted 3-4 times for each transgenic line, the scores combined, and the means in percentage displayed. Chi square-tests (with Yates correction, degrees of freedom  = 1) were conducted to determine significant differences between the lines.

### Confocal microscopy of transgenic *C. elegans* strains and localization of *Ac-*DAF-16::GFP

Dauer larvae were prepared for confocal microscopy on a Zeiss LSM 710 with a 20x/1.0 water immersion objective immediately before image acquisition by transfer into M9 buffer containing 10 mM levamizole. Photographs were taken within 15 min of removal from incubators and exposure to levamizole. Photomicrographs represent?multichannel?λ-stacks recorded between 500-550 nm by a 32-channel spectral detector and reveal both GFP and autofluorescence as different spectra.

## Results

### *Ac-*DAF-16 as substrate of AKT

**Figure 1:**
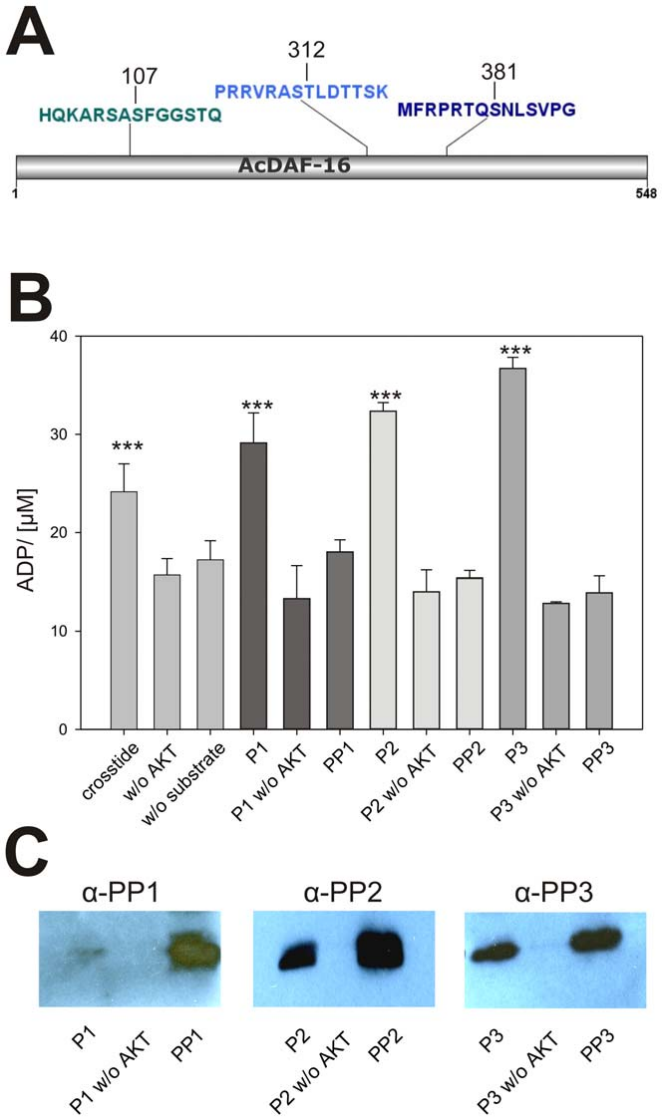
AKT phosphorylates *Ac*-DAF-16 on conserved phosphorylation sites. **A**) Graphical scheme of *Ac*-DAF-16 indicating the localization of the predicted AKT phosphorylation sites at position S107, T312 and T381 and the peptides (P1, P2, P3) of 15 amino acids lengths used in the *in vitro* phosphorylation assay. **B**) *In vitro* phosphorylation assay of *Ac*-DAF-16 peptides by AKT. The means ± SD of the ADP concentration after incubation of rAKT with the *Ac*-DAF-16 peptides in the presence of ATP is shown. Crosstide was used as a control substrate. Negative controls were reactions without AKT as well as phospho-peptides (PP1, PP2, PP3) containing the phosphorylated residue of interest. The experiments were repeated three times. Asterisks represent p- values <0.05 in T-tests between the sample versus controls. **C**) Reactions from the *in vitro* phosphorylation assay were separated by PAGE and blotted on nitrocellulose membrane. Proteins were detected by phospho-specific peptide antibodies as described.

In *C. elegans*, AKT modifies DAF-16 at the four phosphorylation sites (Ser54, Ser240/242, Ser314), thereby creating the binding site for the FTT-2 interaction [14], [30]. We recently showed that *Ac*-DAF-16 was immunoprecipated with *Ac*-FTT-2 from serum-activated L3 lysates, and that the interaction required intact AKT phosphorylation sites at Ser107 and Thr312 [27]. To determine if AKT similarly modified *Ac*-DAF-16, we incubated 3 peptides containing the predicted phosphorylation sites (Fig. 1A) with recombinant AKT in vitro, using ADP formation as an indicator of kinase activity. As shown in Fig. 1B, the ADP concentration in all three reactions was significantly increased in the presence of AKT and the single peptide compared to the negative controls, indicating that the hydrolysis of ATP by AKT kinase occurred. The conserved motif for AKT phosphorylation is RxRXXS/THyd, where x represents any amino acid residue [31], and is present in all three peptides. In addition to these sites, motif recognition software [32] predicted a possible AKT phosphorylation site adjacent to Thr312 at Ser311. However, there was no increase in ADP concentration in kinase reactions using a peptide phosphorylated at Thr312, indicating that Thr312 was the only reactive site on the peptide, and that Ser311 was not phosphorylated by AKT.

The phosphorylation of the peptides by AKT was verified by Western blot analysis using phospho-specific anti-DAF-16 antibodies (Fig. 1C). Phosphorylated peptides were detected in the AKT + ATP + peptide reactions, but not in reactions without the enzyme. Together, these data indicate that *Ac*-DAF-16 is phosphorylated on the conserved residues Ser107, Thr312 and Ser381 by AKT.

### Sub-cellular localization of *Ac-*DAF-16

**Figure 2:**
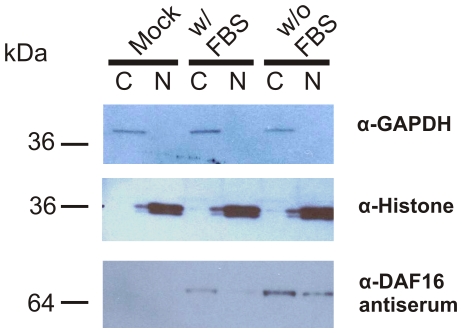
*Ac*-DAF-16 is localized in cytoplasm and, in the absence of IIS, also in the nucleus. Western blot analyses of the cytosolic (C) and the nuclear (N) fractions of HEK293 cells expressing Daf16.4Ba.pCMV.2J are shown. After incubation for 2 days in the presence of 10% FBS, the cells were further cultivated in the medium containing 10% FBS (lane w/FBS) or medium without FBS (lane w/o FBS) for 24 h. MOCK cells were transfected with the empty pCMVtag4 vector and treated as the cells w/o FBS. As controls antibodies against the cytoplasmic enzyme GAPDH and the nuclear histone were used to determine the purity of the fractionation.

Phosphorylation by AKT, together with our previous studies [27] suggested that *Ac*-DAF-16 is regulated by the 14-3-3 dependent shuttling mechanism seen in *C. elegans* [14]. We used a cell culture system to determine if *Ac*-DAF-16 changes sub-cellular compartments in response to IIS stimulation. HEK293 cells expressing full length wildtype *Ac*-DAF-16 were incubated with or without serum, followed by sub-cellular fractionation and Western blotting to determine the location of DAF-16 (Fig. 2). Antibody against GAPDH was used as a marker for the cytoplasmic compartment, and histone H1 for the nuclear compartment. Both marker proteins were detected at similar levels in the appropriate fraction. *Ac*-DAF-16 was detected in both compartments in serum-starved cells, but only in the cytoplasm in fed cells, suggesting that *Ac*-DAF-16 localization is controlled by IIS, similar to *C. elegans* DAF-16.

### Negative regulation of *Ac-*DAF-16 transcriptional activity

**Figure 3:**
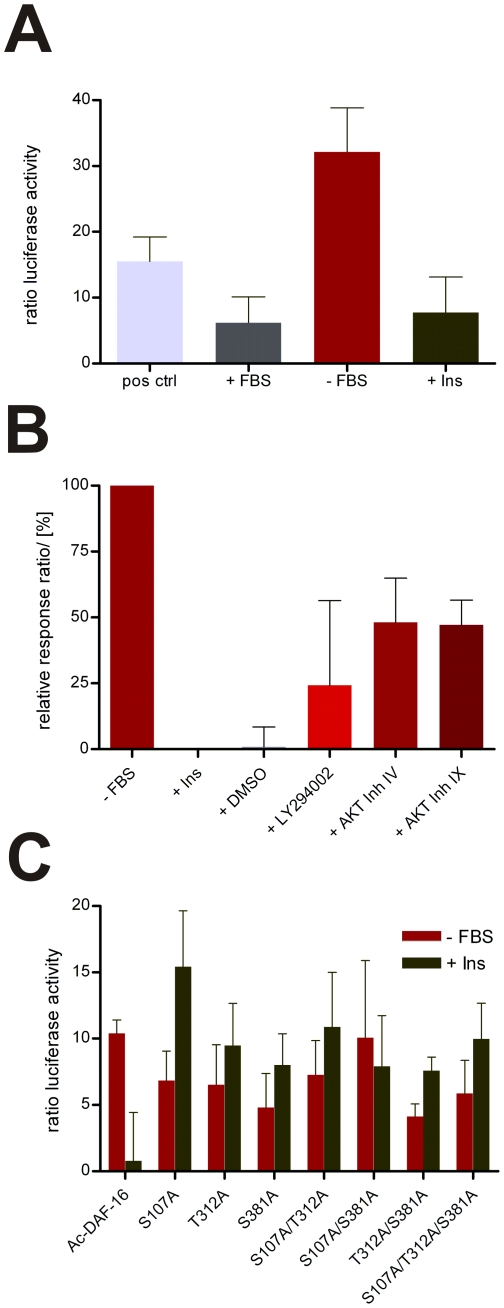
Gene transcription from DAF-16 binding elements driven by *Ac*-DAF-16 is negative regulated by IIS. **A**) Luciferase activity of insulin sensitive HepG2 expressing full-length *Ac*-DAF-16 treated with serum (+ FBS), without serum (−FBS) and insulin (+Ins). **B**) Relative luciferase activity of cells cultivated without serum, treated with insulin and subsequent treated with IIS inhibitors. LY294009 is a specific inhibitor of PIP3K and AKT Inhibitor IV and IX specifically inhibit AKT. The luciferase activity is depicted relative to starved cells (100% gene expression) and to insulin treated cells (0% expression). **C**) Comparison of DBE-driven transcription of *Ac*-DAF-16 wildtype and AKT-phospho-mutants when cells were treated as described in A).

To analyze the function and regulation of *Ac*-DAF-16, we assessed its transcriptional activity from a DAF-16 derived promoter element under the influence of serum and IIS. Insulin sensitive HepG2 cells were co-transfected with a construct encoding full-length *Ac*-DAF-16, and a reporter construct containing 6 copies of the DAF-16 binding element (DBE) upstream of the firefly luciferase gene. A construct encoding *Renilla* luciferase under the CMV promoter was also included as an internal control, and a plasmid encoding constitutively expressed luciferase served as positive control. The ratios of the luciferase activity of cells incubated in medium with serum, serum-starved cells, and cells stimulated with insulin following starvation are depicted in Figure 3A. Luciferase expression was five-fold higher in starved cells than serum-fed cells, whereas addition of insulin to starved cells depressed luciferase expression to the level of serum-fed cells. Next, inhibitors of IIS were tested for their effects on insulin stimulated cells. As shown in Figure 3B, AKT inhibitors IV and IX (Calbiochem) increased normalized luciferase expression 48% and 47%, respectively, and the PI3K inhibitor LY294002 increased expression by 24%. These data confirm that *Ac*-DAF-16 drives transcription from the DBE in the absence of IIS, and that IIS negatively regulates DAF-16 mediated reporter transcription.

In *C. elegans*, AKT phosphorylates DAF-16 on conserved sites in response to IIS, creating a binding site for the shuttle protein 14-3-3. The bound DAF-16 is translocated from the nucleus to the cytoplasm, resulting in negative regulation of DAF-16 transcription. To determine the mechanism by which insulin negatively regulates *Ac*-DAF-16 in cells, the effect of phosphorylation site null mutants on DAF-16 driven transcription was tested. *Ac*-DAF-16 constructs containing single, double and triple mutants of the three AKT phosphorylation sites were co-transfected with the reporter and *Renilla* control plasmids in HepG2 cells. As shown in Figure 3C, wildtype *Ac*-DAF-16 drove transcription in the absence of serum, whereas insulin inhibited transcription. As expected, all of the AKT site mutants were able to drive transcription from the DBE in the absence of serum. However, mutation of any of the phosphorylation sites to Ala prevented inhibition of transcription by insulin. The higher read-out for mutated *Ac*-DAF-16 when insulin was added could be due to longer lasting mutated *Ac*-DAF-16 which are not degraded as fast as wildtype *Ac*-DAF-16 [33]. This indicates that insulin-induced inhibition of transcription is mediated by phosphorylation of the consensus AKT sites, and that mutation of any of the sites prevents inhibition of transcription by insulin.

### Rescue of the dauer phenotype in *C. elegans daf-2; daf-16* strain expressing *Ac-*DAF-16

**Figure 4:**
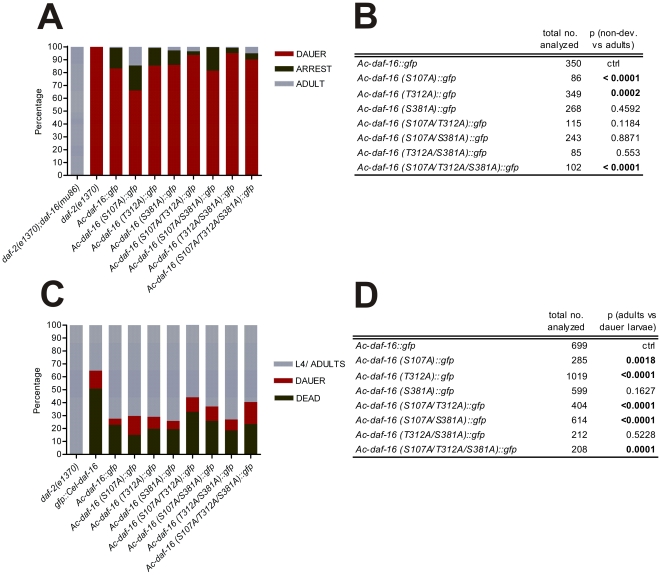
*Ac*-DAF-16 complements missing endogenous *Ce* –DAF-16 in *C. elegans* double mutant *daf-2; daf-16*. **A**) Rescue of dauer defective phenotype of *C. elegans daf-2;daf-16* mutants by *Ac*-DAF-16 wildtype and AKT phosphorylation mutants at 25°C. **B**) Chi-square analysis of A) showing significant p-values in bolt. **C**) Recovery of SDS isolated dauers complemented with *Ac*-DAF-16 wildtype and AKT phospho-mutants at 16°C. **D**) Chi-square analysis of C.

As genetic manipulation of hookworms is currently not possible, we used *C. elegans* as a surrogate to study the function of *Ac*-DAF-16 *in vivo* during dauer entry and exit. Whereas *daf-2(e1370)* mutants reared at the restrictive temperature form 100% dauers, the dauer phenotype is completely suppressed in *daf-2(e1370); daf-16(mu86)* double mutants. Thus, rescue of the dauer phenotype in *daf-2(e1370);daf-16(mu86)* double mutants provides a convenient assay for *Ac*-DAF-16 function in *C. elegans*. The *C. elegans* dauer defective double mutant strain *daf-2(e1370);daf-16(mu86)* was complemented with constructs expressing either *Ac*-DAF-16 wildtype or AKT site phospho-mutants by microinjection and incubated at the *daf-2* restrictive temperature of 25°C, and stable extrachromosomal strains established. Transgenic animals were selected, their progeny allowed to hatch after which they were shifted to restrictive temperature; 4 days later animals were scored. The majority (83%) of the offspring developed into dauers, 16% were arrested larvae and less then 1% developed into the adult hermaphrodites, indicating that *Ac*-DAF-16 can partially rescue the dauer phenotype (Fig. 4 A and B). Single mutation on S381 and double mutations on two of the AKT phosphorylation sites S107, T312 and S381 had no effect on the capability of *Ac*-DAF-16 to rescue the dauer phenotype. Only worms expressing the *Ac*-DAF-16 single mutants S107A and T312A or the triple mutant showed statistically significant development to the adult (p-values<0.0002; Fig. 4 A and B).

### Dauer exit of transgenic *C. elegans daf-2;daf-16* dauers

We hypothesized that *Ac*-DAF-16 is one of the major regulators of exit from the developmentally arrest L3 stage during hookworm infection of the host. Therefore, we asked whether transgenic *C. elegans* dauers are able to exit dauer. We predicted that *daf-2;daf-16* dauers expressing wildtype *Ac*-DAF-16 would exit the dauer stage and develop into adults when returned to permissive temperatures. We would further expect that *daf-2;daf-16* dauers expressing AKT phosphorylation mutants of *Ac*-DAF-16 might fail to exit dauer, depending on the importance of the mutated sites for 14-3-3 binding. For example, triple mutated *Ac*-DAF-16 would not be phosphorylated by AKT and therefore dauers expressing the *Ac*-DAF-16 triple mutant would not be expected to exit dauer. Single mutations, however, might have a more subtle effect, and some dauers may be able to progress in development.

To determine the effect of phosphorylation site mutations on dauer exit, we isolated dauer larvae after 4 days incubation at restrictive temperature, followed by incubation at the permissive temperature of 16°C. All of the control *daf-2(e1370)* dauers recovered at permissive temperature. Surprisingly, most of dauers developed to L4/adults in transgenic lines expressing *Ac*-DAF-16 (Fig. 4C and D). However, fewer of the dauers carrying the *Ce*-DAF-16 transgene formed adults; about 51% of dauer larvae died, 14% remained dauers and only 35% were able to resume development. Most of the dauer larvae expressing wildtype *Ac*-DAF-16 exited dauer and developed into adults (72%), whereas 23% died and only 5% persisted as dauer larvae. A similar outcome was seen for the *Ac*-DAF-16 single mutant S381A (74% adults, 6% dauer larvae) and the *Ac*-DAF-16 double mutant T312A/ S381A (73% adults, 8% dauer larvae). A significantly higher fraction remained as dauer larvae when *Ac*-DAF-16 was mutated on the first and/or second, first and third phosphorylation site, and on all three phosphorylation site. We found 15% of the larvae were dauers in the S107A mutants, 9% in the T312A mutants, and 11% each in the S107A/T312A and S107A/S381A mutant. When all three sites were mutated, 17% of the larvae remained dauers, 23% died and 60% developed to the L4/adult stage. The statistical analysis indicated that phosphorylation on S107 and T312 are the most important residues for AKT regulation of *Ac*-DAF-16. Both residues were previously shown to be required for 14-3-3 binding [27]. However, most dauer larvae recovered even when all three AKT regulation sites were mutated. This suggests that an AKT-independent mechanism is involved in the regulation of *Ac*-DAF-16 in *C. elegans* during the dauer exit. It further suggests that hookworm L3 might exit arrest similarly by an AKT-independent mechanism.

### Localization of *Ac-*DAF-16 expressed in *C. elegans* during dauer and dauer exit

Regulation of DAF-16 by IIS is associated with shuttling from the nucleus to the cytoplasm. However, the majority of transgenic larvae entered and exited the dauer stage, even without phosphorylation-competent sites. Therefore, we asked where *Ac*-DAF-16 is localized during dauer and when the larvae exit the dauer stage, and whether mutations on the AKT phosphorylation sites affect its localization. Using confocal microscopy, the localization of wildtype and phosphorylation site mutant *Ac*-DAF-16::GFP fusion proteins was determined in dauers and recovering dauers 16 h after downshift to 16°C. At this time, the larvae were recovering, but remained morphologically dauers.

**Figure 5:**
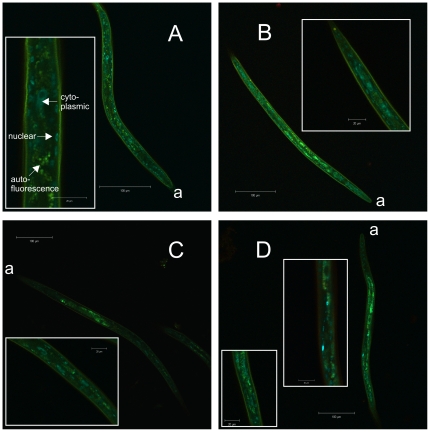
Cellular localization of *Ac*-DAF-16 wildtype and single AKT-phospho-mutants in dauer larvae. Pictures are lambda stack images from transgenic *daf-2;daf-16* dauers, expressing fusion constructs of **A**) *Ac*-DAF-16 wildtype, single mutants **B**) *Ac*-DAF-16 (S107A), **C**) *Ac*-DAF-16 (T312A), **D**) *Ac*-DAF-16 (S381A). “a” indicates the anterior end.

**Figure 6:**
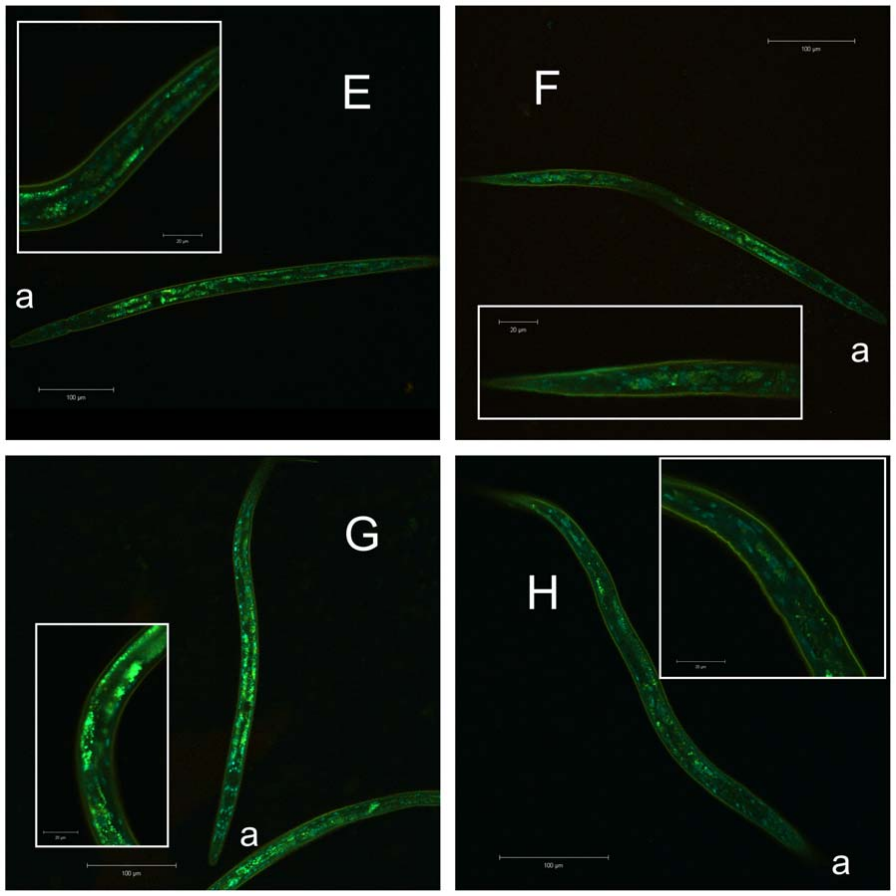
Cellular localization of *Ac*-DAF-16 double and triple AKT-phospho-mutants in dauer larvae. Pictures are lambda stack images from transgenic *daf-2;daf-16* dauers, expressing fusion constructs of double mutants **E**) *Ac*-DAF-16 (S107A/T312A), **F**) *Ac*-DAF-16 (S107A/S381A), **G**) *Ac*-DAF-16 (T312A/ S381A) and **H**) *Ac*-DAF-16 triple mutant. “a” indicates the anterior end.

**Figure 7:**
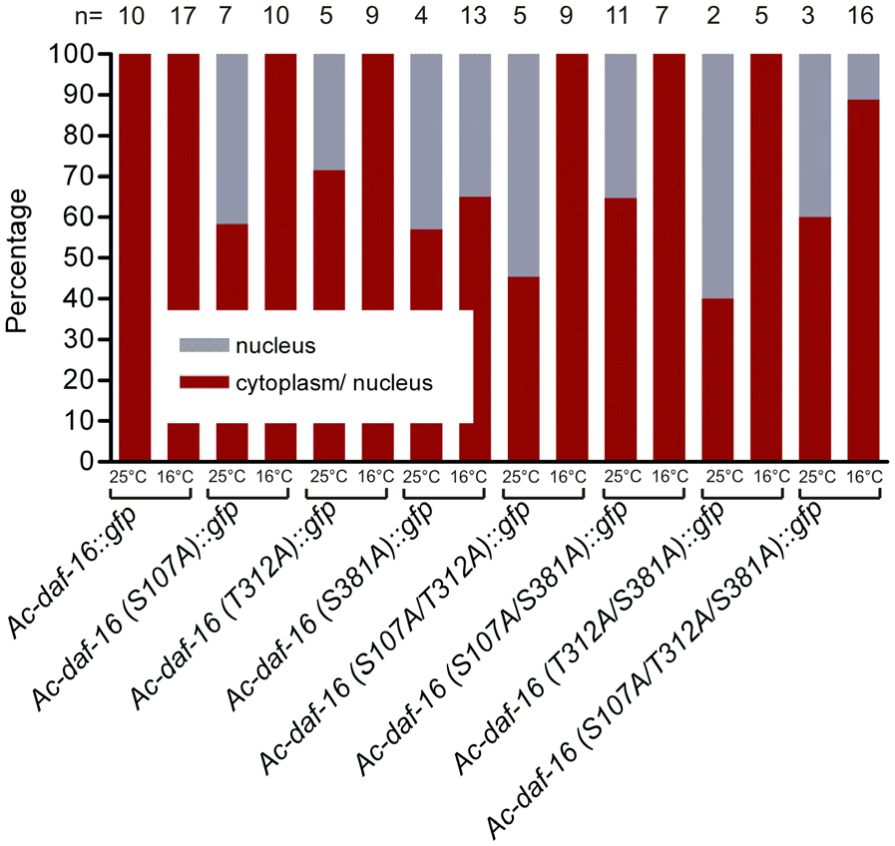
Quantified sub-cellular localization of *Ac*-DAF-16 wildtype and AKT-phospho-mutants in dauer larvae. Dauer larvae showing nuclear and nuclear/cytoplasmic *Ac*-DAF-16::GFP were quantified during dauer and following induction of dauer exit.

In dauer larvae incubated at 25°C, *Ac*-DAF-16 wildtype was localized primarily in nuclei of hypodermal and body wall muscle cells, as well as some intestinal cells; these cells also had very low cytoplasmic levels of GFP. Wildtype *Ac*-DAF-16 was never observed exclusively in the nuclei of any cells in dauers (Fig. 5). When dauer larvae were downshifted to 16°C, wildtype *Ac*-DAF-16 was expressed in the same cells, predominantly in the nuclei, but a higher proportion was localized in the cytoplasm. Single or multiple mutations on S107, S381 and T312 in *Ac*-DAF-16 did not alter the localization pattern in the different cell types (Fig. 5 and 6), but the mutations did affect sub-cellular localization. These differences were quantified by counting dauers with purely nuclear DAF-16::GFP signal and those with DAF-16::GFP in both cellular compartments. Exclusively cytoplasmic expression of either wildtype or mutated *Ac*-DAF-16 was never observed. As shown in Figure 7, *Ac*-DAF-16 S107A was more frequently localized in the nuclei of dauer larvae, and larvae with exclusively nuclear localization were observed. Induction of dauer exit shifted the localization towards the cytoplasmic compartment so that *Ac*-DAF-16 (S107A) was found in both compartments in all worms. A similar response was observed in dauers expressing *Ac*-DAF-16 single mutant T312A and the double mutants (S107A/T312A, S107A/S381A and T312A/ S381A) during dauer and dauer exit. Dauers expressing the single *Ac*-DAF-16 mutation S318A and the triple mutant showed a different pattern. In these cases, DAF-16 remained in the nucleus in a significant number of dauers during dauer exit, although slightly less so in the triple mutant *Ac*-DAF-16. These data indicate that the S381 site can mediate nuclear exclusion during dauer recovery.

## Discussion

Recent publications from our lab and others support the hypothesis that recovery from developmental arrest by the hookworm and other parasitic nematode infective stages is regulated by IIS [27], [29], [34], [35], similar to recovery from dauer in *C. elegans* [36]. Here we provide further evidence supporting a role for IIS in hookworm L3 recovery, in addition to evidence indicating that another, as yet undefined, mechanism is also involved. Using cell-based approaches and *in vitro* assays, we demonstrated that hookworm DAF-16 is a downstream target of IIS and a substrate of AKT kinase. Sub-cellular fractionation of transfected cell lines indicated that a fraction of nuclear localized *Ac*-DAF-16 in starved cells is shuttled to the cytoplasm in response to serum, and *Ac*-DAF-16-driven transcription from the conserved DBE was sensitive to insulin in cell culture. Together, these data suggest that IIS mediates negative regulation of *Ac*-DAF-16. Furthermore, complementation of *C. elegans* dauer defective mutants with *Ac*-DAF-16 restored the dauer phenotype, confirming that *Ac*-DAF-16 is orthologous to *Ce*-DAF-16, and can function in *C. elegans* dauer formation in the absence of functional endogenous DAF-16.

While dauer formation has been a useful paradigm for framing questions about hookworm developmental signaling, dauer recovery is a more relevant process to the resumption of development that occurs when hookworm L3 infect a permissive host. However, there have been few investigations of dauer recovery reported in *C. elegans*. For the first time, we examined the role of a heterologous DAF-16 in recovery from dauer arrest using wildtype and phospho-null AKT site mutants of hookworm DAF-16 in transgenic dauer larvae. Transgenic *C. elegans* dauers expressing wildtype *Ac*-DAF-16 recovered from dauer when shifted to permissive temperature, as would be expected for a DAF-16 ortholog. Surprising, however, dauers expressing the phospho-null mutant *Ac*-DAF-16 also recovered, indicating that the intact, phosphorylation-capable AKT sites were not required for dauer recovery. This also suggests that a mechanism other than AKT/14-3-3 mechanism can mediate dauer recovery in *C. elegans*, and by extension, hookworms.

We also examined the localization of GFP labeled *Ac*-DAF-16 during dauer and dauer recovery. In general, the tissue expression pattern of transgenic *Ac*-DAF-16 in the *C. elegans daf-2;daf-16* mutants conformed to expression of *Ce*-DAF-16 under the *pdaf16α* promoter, namely expression in hypodermis, intestine, body wall muscles and neurons [8], [34]. Dauers expressing wildtype *Ac*-DAF-16 had some cells that had exclusively nuclear localized DAF-16, but no worms were found that had nuclear expression in all cells. This was similar to our cell culture results, in which *Ac*-DAF-16 was found distributed between both the nuclear and cytoplasmic compartments in starved cells, the equivalent of dauers in that they lack significant levels of IIS. This mixed distribution suggests that some shuttling is occurring even under low insulin signaling conditions. Interestingly, 40–60% of transgenic dauers expressing *Ac*-DAF-16::GFP with any phospho-null AKT site mutation showed exclusively nuclear localization of DAF-16, suggesting that basal levels of 14-3-3 shuttling require phosphorylation of these sites in the dauer.

During recovery, localization of wildtype *Ac*-DAF-16 shifted from the nucleus to the cytoplasm, in accordance with AKT phosphorylation and the 14-3-3 shuttle mechanism mediated by IIS. Similarly, all of the transgenic worms expressing exclusively nuclear localized phospho-null *Ac*-DAF-16 underwent a shift to mixed cytoplasmic and nuclear expression with the exception of the S381A mutant, in which only 8% of the worms switched to mixed localization. Approximately 11% of the triple mutants also retained DAF-16 in their nuclei. This indicates a role for S381 in translocation of DAF-16 to the cytoplasm during dauer recovery. The requirement for an intact S381 is not absolute, however, as all double mutants containing S381A showed nuclear localization during recovery. This suggests that at least two independent mechanisms control DAF-16 translocation during recovery. One mechanism requires either intact S107 or T312 sites on DAF-16 for shuttling, whereas the other requires an intact S381 site. AKT phosphorylation sites S107 and T312, but not S381, were shown to be required for interaction with hookworm 14-3-3 [27], suggesting that the S107/T312-dependent mechanism may represent the canonical AKT/14-3-3 shuttle [14], and that S381 mediates a 14-3-3-independent translocation of DAF-16 from the nucleus to the cytoplasm during dauer recovery. Previous publications suggested already that shuttling is not a requirement to silence DAF-16/FoxO transcriptional activity as shown in cell based assays [14], [37], but a mechanism was not defined.

As all of the worms expressing phospho-null *Ac*-DAF-16 recovered from dauer, even those lacking site S381 and the triple mutants, recovery from arrest does not require phosphorylation of the known AKT sites in *Ac*-DAF-16. Furthermore, while *Ac*-DAF-16 exits the nucleus during recovery, translocation is not essential, as most worms expressing S381 phospho-null *Ac*-DAF-16 recover from dauer despite retention of DAF-16 in the nucleus. This suggests that a molecular mechanism independent of, or in addition to, AKT can negatively regulate DAF-16 activity in response to IIS. Additional outputs of AKT or other IIS kinases might indirectly regulate DAF-16.

Numerous studies describing the role of DAF-16 and IIS in *C. elegans* dauer formation and aging have been reported. DAF-16 expression in neurons is required to restore the dauer phenotype in dauer defective *daf-2;daf-16* mutants, whereas intestinal expression is required for increased longevity [28]. Expression of wildtype and mutated versions of *Ac*-DAF-16 were sufficient to restore the dauer phenotype in dauer defective *daf-2;daf-16* mutants, suggesting neuronal expression in transgenic dauers during recovery despite our inability to confirm this visually. However, we could not identify a comparable study addressing the localization of DAF-16 during dauer exit. In transgenic *Strongyloides stercoralis* L1, Castelletto et al showed that phospho-null mutants of the DAF-16 ortholog *Ss*-FKTF-1 are trapped inside the nucleus because AKT/14-3-3 binding sites are missing [35], but the localization of *Ss*-FKTF-1 during dauer exit was not addressed. Therefore, we showed for the first time that DAF-16 of parasitic origin shuttles from the nucleus to cytoplasm during dauer exit in a process that is not exclusively dependent on AKT/14-3-3 regulation. Complementation assays of *C. elegans* double mutants with *Ss-Fktf-1* also restored the dauer phenotype [34], but the cellular localization of *Ss*-FKTF-1 was not reported. In the same study, complementation with the homogenous *Ce*-DAF-16 restored the dauer phenotype in a lower percentage of worms than complementation with the heterologous parasite transcription factor [34]. We saw similar results in the dauer exit assays, a higher proportion of dauers expressing *Ac*-DAF-16 exited than *Ce*-DAF-16 expressing dauers. The reason for this anomaly is unknown, but may be construct- related.

Cahill et al reported 14-3-3 dependent and independent regulation of DAF-16 ectopically expressed in HepG2 cells [14]. Our cell based approach, using *Ac*-DAF-16 wildtype and AKT site mutants, confirms the results and showed that even AKT/14-3-3 null mutants were insulin sensitive. Furthermore, all cells expressing *Ac*-DAF-16 variants reacted much more strongly to insulin than to FBS, suggesting regulation of *Ac*-DAF-16 is highly complex, and that regulation in response to other growth factors in addition to insulin contained in FBS might be involved as well. Also, cell based assays involving growth factor treatment to assess DAF-16 regulation must be interpreted carefully, as ubiquitinylation processes have been reported to lead to degradation of FoxO proteins [33], [38]. However, in addition to the cell based assay, our dauer rescue and recovery data strongly suggest that an additional, AKT/14-3-3-independent mechanism regulates *Ac*-DAF-16.

As DAF-16 is a convergence point for several developmental pathways, it is also regulated by IIS dependent and independent outputs from these pathways [39]–[46]. These include co-translational (i.e. myristylation) and post-translational modifications like acetylation, deacetylation, methylation, dephosphorylation, and phosphorylation by kinases other than AKT. The role of these regulatory mechanisms in recovery from dauer and L3 arrest are unknown, but it is possible that these may play a more significant role in regulating hookworm L3 DAF-16 than in the *C. elegans* dauer stage. Further investigations are necessary to shed light on the regulation of the hookworm L3 arrest and the role of IIS, as well as IIS independent mechanisms in the re-activation of infective, developmentally arrested hookworms.
